# Peripheral Neuropathy as a Component of Skeletal Disease in Diabetes

**DOI:** 10.1007/s11914-019-00528-8

**Published:** 2019-08-07

**Authors:** Alec T. Beeve, Jennifer M. Brazill, Erica L. Scheller

**Affiliations:** 1 Department of Medicine, Division of Bone and Mineral Diseases, Washington University, 660 South Euclid Avenue, Saint Louis, MO 63110 USA; 2 Department of Biomedical Engineering, Washington University, 6201 Forsyth Blvd, Saint Louis, MO 63105 USA; 3 Department of Cell Biology and Physiology, Washington University, 660 South Euclid Avenue, Saint Louis, MO 63110 USA

**Keywords:** Diabetes, Neuropathy, Metabolic bone disease, Fracture, Microvascular disease, Marrow adiposity, Marrow fat

## Abstract

**Purpose of Review:**

The goal of this review is to explore clinical associations between peripheral neuropathy and diabetic bone disease and to discuss how nerve dysfunction may contribute to dysregulation of bone metabolism, reduced bone quality, and fracture risk.

**Recent Findings:**

Diabetic neuropathy can decrease peripheral sensation (sensory neuropathy), impair motor coordination (motor neuropathy), and increase postural hypotension (autonomic neuropathy). Together, this can impair overall balance and increase the risk for falls and fractures. In addition, the peripheral nervous system has the potential to regulate bone metabolism directly through the action of local neurotransmitters on bone cells and indirectly through neuroregulation of the skeletal vascular supply.

**Summary:**

This review critically evaluates existing evidence for diabetic peripheral neuropathy as a risk factor or direct actor on bone disease. In addition, we address therapeutic and experimental considerations to guide patient care and future research evaluating the emerging relationship between diabetic neuropathy and bone health.

## Introduction

In the nineteenth century, a fundamental link was established between neuropathy and skeletal disease. In 1868, Jean-Martin Charcot, now considered a pioneer in the emerging field of neuroskeletal biology, reasoned that the pathogenesis of degenerative bone and joint disease was secondary to syphilitic damage to the spinal cord [1]. The proposed etiology for the disease, now referred to as Charcot neuroarthropathy, attracted debate and a series of investigations that together suggest a trifold pathological process mediating the complex relationships between nerves and bone, including altered loading and microdamage (neurotraumatic), impaired local neurotransmitter release (neurotrophic), and reduced neural regulation of bone blood flow (neurovascular) [1]. With the rise of antibiotics and resulting decline of syphilis, diabetes has now emerged as the leading cause of neuroarthropathy [2]. Additionally, patients with type 1 and type 2 diabetes (T1D and T2D) develop significant changes in bone even in the absence of Charcot joint disease, which likewise increase fracture risk and decrease quality of life [3]. Recent investigations suggest that the same neuropathic processes responsible for neuroarthropathy may also contribute to the development of diabetic bone disease. Subsequent findings could not only aid in the prevention of debilitating neuroarthropathy and fracture but also provide an avenue to further study the relationships between nerve and bone health.

## Diabetic Bone Disease

Though both diseases lead to increased fracture risk, the severity and nature of bone disease differs between patients with T1D and T2D. T1D is an early onset, autoimmune disorder that results in the destruction of pancreatic beta cells and systemic insulin deficiency [3]. Patients with T1D have decreased bone turnover [4] and a decrease in bone mineral density (BMD) which does not fully explain the ~ 6-fold increase in fracture risk [5, 6]. When present, changes in skeletal microarchitecture in adults with T1D include decreased trabecular bone volume fraction, decreased cortical thickness, and increased bone size as measured by cross-sectional area and periosteal circumference [7, 8, 9••, 10•, 11]. In one study, the persistence of cortical and cross-sectional changes in adulthood was dependent on childhood onset of T1D [9••]. Indeed, several studies suggest that children and adolescents with T1D are at risk for decreased bone mass during development, which may prevent acquisition of optimal peak bone quantity, quality, and strength [12, 13••]. Epidemiologic studies further indicate that early changes in T1D bone contribute to significant increases in fracture risk throughout the lifespan [14].

On the other hand, T2D is associated with a variety of genetic and environmental factors leading to insulin resistance, in which the biological efficacy of insulin is impaired [15]. As with T1D, T2D patients have decreased bone turnover and increased fracture risk, though both to a lesser extent [4–6]. Thirteen high-resolution peripheral quantitative computed tomography (HR-pQCT) studies of adult T2D bone have demonstrated that, despite significant heterogeneity, T2D is generally associated with limited to modest deficits in cortical bone density and area and, unlike T1D, relative preservation of or improvements in trabecular architecture [8, 16•, 17–19, 20••, 21–23, 24•, 25–27]. In a recent study, trabecular improvements were limited to the early stages of T2D (< 10 years) [26], which may be related to increased loading due to larger body size or early hyperinsulinemia, prior to the onset of diabetic complications and co-morbidities. Though variable, an increase in cortical porosity is one of the more consistent findings by HR-pQCT in patients with T2D [16•, 17, 21], with a trend toward increased pore size even when compared directly to patients with T1D [8]. Increased cortical porosity is more prevalent in patients with a previous history of fracture [25, 27], with poor glucose control [18], or in the presence of microvascular disease [20••].

The pathogenesis of bone disease in diabetes likely represents several underlying processes which converge on bone in a disease-, stage-, age-, and even patient-specific manner (reviewed in [3, 28, 29]). This is a substantial clinical problem. Within the general population, ~ 8–20% of older adult patients die within 1 year of hip fracture and > 50% never regain functional independence [30–33]. These outcomes are even worse for patients with diabetes, with higher rates of post-operative complications, longer hospital stays, substantial increases in mortality, greater frequency of pain/depression, and higher likelihood of nursing home placement [33–38]. Identification of novel, individualized risk factors, and mechanisms underlying diabetic bone disease and fracture risk is necessary to enhance treatments, thus prolonging health span in aging patients with diabetes.

## Diabetic Neuropathy

Diabetic peripheral neuropathy (DPN) is a common complication of both T1D and T2D [39, 40] and a potential contributor to bone disease. About half of all diabetic patients are expected to develop DPN over the course of the disease, although studies disagree on whether prevalence is higher in T1D [41, 42], T2D [43••, 44], or similar in both [45]. DPN most often originates bilaterally in the long axons of the lower limbs and progresses proximally from the feet, affecting sensory, motor, and autonomic nerve fibers [46–48]. Degeneration of small sensory nerves is often, but not always, associated with painful, spiking sensations; this is followed by degeneration of large myelinated sensory fibers and associated numbness [49]. In addition to sensory dysfunction, patients with DPN may also present with other symptoms indicative of motor or autonomic dysfunction. For example, diabetic motor neuropathy may contribute to muscular atrophy [50] and autonomic neuropathy may precipitate cardiovascular, gastrointestinal, sudomotor, or urogenital dysfunction [48].

Like diabetic bone disease, DPN is a multifactorial complication with an evolving list of risk factors (reviewed in [46, 47]). While gaps remain in our understanding of its pathogenesis, hyperglycemia and dyslipidemia are indispensable to DPN onset and progression. These two factors, in concert with many others, are currently hypothesized to result in an acute metabolic stage (reversible) and a chronic structural stage (irreversible) of diabetic neuropathy [51]. In the acute metabolic stage, excess intracellular glucose is metabolized by one of three pathways: glycolysis, the hexosamine pathway, or the polyol pathway. Activation of these pathways results in the initiation of inflammatory cascades and the formation of reactive oxygen species, which can induce oxidative stress and disrupt nerve energy supply. Polyol pathway activation results in reduced Na^+^/K^+^-ATPase activity and accumulation of Na^+^ in the axon, impairing nerve excitability and slowing nerve conduction velocities [52–54].

In the chronic stage of DPN, degeneration of small and large nerve axons can be observed in the peripheral nervous system via teased fiber analysis, sural nerve biopsies, and skin punch biopsies [51, 55–57]. Axonal swelling from Na^+^ accumulation disrupts connective junctions between Schwann cells and their associated axons [58]. Chronic hyperglycemia leads to formation of advanced glycation end products in neurons and Schwann cells, which can impair protein and cellular function, evoke inflammatory cascades, and result in additional oxidative stress [59, 60]. Free fatty acids can damage Schwann cells directly and promote insulin resistance, and oxidation of lipoproteins and cholesterol can further increase oxidative stress and induce neuronal apoptosis [61–63]. In addition to direct nerve damage, the mechanisms described above also contribute to development of microvascular disease (MVD) that may lead to neural ischemia [64]. As structural damage proceeds, injured nerves may attempt to repair themselves, overexpressing persistent sodium channels, and releasing neuropeptides that may cause ectopic firing perceived as pain [54, 65], but ultimately, regeneration fails due to intrinsic neuronal deficits, Schwann cell dysfunction, and an unfavorable extracellular environment [46, 47]. Insurmountable chronic nerve injury leads to DPN, initially posing a threat to the limb, but also increasing the risk of death as it progresses proximally, affecting cardiovascular autonomic nerve fibers [46]. In addition to the heart, the impact of DPN on all innervated organs, including bone, needs to be considered in order to provide the most comprehensive care to patients with diabetes.

## Clinical Relationships Between Diabetic Bone Disease and Neuropathy

To evaluate clinical associations between diabetic bone disease and DPN, a database search was performed on PubMed and Google using the following search keywords: “diabetes,” “neuropathy,” “microvascular disease,” “microangiopathy,” “bone mineral density,” “bone microarchitecture,” “bone serum markers,” “fracture,” and “bone healing complications.” All studies with human patients were evaluated. Additional articles were obtained from reference lists. Individual articles from meta-analyses and systematic reviews were included, but not the meta-analyses themselves. Records only addressing retinopathy or nephropathy or relating to falls were excluded from our qualitative synthesis, as they are not the focus of this review. A summary of the articles and their findings is presented in Table 1.

**Table 1:** Summary of clinical studies associating diabetic neuropathy (or microvascular disease) and bone disease, fracture or healing complications

Study	Sex	Age	Sample size	Diabetes type	Assessed relationship	Nerve assessment method	Bone assessment method	Relationship? (Y/N)	Findings^a^	Reference
1	M/F	32 ± 8	326	T1D	Neuropathy	PROs, QST, reflex tests	DEXA	Y	Low BMD associated with chronic diabetes complications, including DPN. IV	Eller-Vainicher et al. [66]
2	M/F	18–54	90	T1D	Neuropathy	NR	DEXA	Y	Patients with low BMD had a higher incidence of DPN and retinopathy. IV	Kayath et al. [67]
3	M	57 ± 6	63	T1D	Neuropathy	PROs, VPT, reflex tests	DEXA, QUS	Y	DPN is an independent risk factor for reduced BMD in T1D. IV	Rix et al. [68]
4	M/F	19–72	71	T1D	Neuropathy	VPT, reflex tests, PROs, sudomotor response, HRV	DPA	Y	Relationship between decreased femoral neck BMD and DPN exists, but not in distal limb or lumbar spine. IV	Forst et al. [69]
5	M/F	20–80	329	T1D	Neuropathy (autonomic)	HRV	DEXA	Y	Reduced BMD in femoral neck associated with autonomic neuropathy. IV	Hansen et al. [70]
6	M/F	20–56	94	T1D	MVD	NR	DEXA	Y	MVD contributes to progression of diabetic osteopenia. IV	Munoz-Torres et al. [71]
7	M	43 ± 5	72	T1D	Neuropathy	NR	DEXA	N	No difference in BMD between patients with and without DPN. IV	Miazgowski et al. [72]
8	M/F	40 ± 9	79	T1D	Neuropathy	NR	DEXA	N	No association between DPN and low BMD. IV	Miazgowski and Czekalksi [73]
9	M/F	62 ± 11	65	T2D	Neuropathy	Monofilament, VPT	DEXA	N	No relationship between heel BMD and DPN, but it is an independent predictor of vascular calcification. IV	Singh et al. [74]
10	M/F	45 ± 11/ 39 ± 9	66	T1D	Neuropathy (autonomic)	HRV	DEXA	N	Autonomic dysfunction does not impact BMD. IV	Maser et al. [75]
11	F	35–55	304	T1D	Neuropathy	MNSI, monofilament, VPT	DEXA, QUS	N	In multivariate models, no relationship between diabetic complications and BMD/QUS. IV	Strotmeyer et al. [76]
12	M/F	11–70	350	T1D/T2D	Neuropathy	EMG, NCV	DEXA, QUS	N	No statistically significant relationship between BMD, QUS and DPN. IV	Chakrabarty et al. [77]
13	M/F	62 ± 12	66	T1D/T2D	Neuropathy	MNSI, VPT	DEXA, QUS, serum	N	Higher deoxypyridinoline in patients with DPN versus those without, but no difference in BMD or QUS. IV	Piaggesi et al. [78]
14	M/F	60 ± 5	49	T1D/T2D	Neuropathy	VPT	DEXA, serum	N	No difference in markers or BMD between patients with and without DPN; predominately type 2 cohort. IV	Christensen et al. [79]
15	M/F	40–80	120	T2D	Neuropathy	Monofilament, reflex test, NCV (upper/lower)	DEXA, serum	Y, males only	After multivariate adjustments, C-telopeptide and P1NP are significantly lower in diabetic patients with DPN. IV	Rasul et al. [80]
16	M/F	19–61	41	T1D/T2D	Neuropathy	Reflex tests, HRV, PROs, ulcer history	X-ray	Y	Cortical bone mass in feet and hands reduced in severe DPN; predominately T1D cohort. IV	Cundy et al. [81]
17	M/F	59–67	194	T1D/T2D	MVD	NR	Serum	Y	MVD associated with increased p-CTX in T1D and p-ucOC in T2D. IV	Starup-Linde et al. [4]
18	M/F	70 ± 8	46	T1D/T2D	Neuropathy	Monofilament, VPT, temperature, Neurotip (pain sensation), HRV	pQCT	N	No relationship found between patients with and without DPN; trending increase in trabecular BMD; predominately type 2 cohort. IV	Barwick et al. [82]
19	M/F	46 ± 12	110	T1D	MVD	VPT, monofilament, reflex tests	HR-pQCT	Y	Compared to controls MVD+, T1D MVD+ have lower trabecular and cortical volumetric BMD, reduced cortical thickness, and increased periosteal circumference; compared to T1D MVD-, T1D MVD+ have decreased trabecular volumetric BMD and thickness. IV	Shanbhogue et al. [7]
20	M/F	~40–72	102	T2D	MVD	VPT, monofilament, reflex tests	HR-pQCT	Y	T2D MVD+ have trends toward increased cortical porosity, decreased cortical thickness, decreased cortical volumetric BMD, and increased trabecular bone mass, not seen in controls or T2D MVD-. IV	Shanbhogue et al. [20]
21	M/F	40–75	410	T2D	MVD	VPT	HR-pQCT	N, but possible trend	T2D MVD+ did not significantly differ from T2D MVD-, except increased cortical porosity and cortical pore volume in radius (but not tibia) in MVD+. IV	de Waard et al. [18]
22	M	65–99	2,798,309	T2D	Neuropathy	ICD codes	Fracture	Y	DPN explains 21% of T2D-associated fracture risk. III-2	Lee et al. [83••]
23	M/F	NR	24,605	T1D	Neuropathy	ICD codes	Fracture	Y	30–40-fold increase in hospitalization rate for hip fracture with DPN. III-2	Miao et al. [84]
24	M/F	32–54	600	T1D	Neuropathy	PROs, QST, reflex tests	Fracture	Y	Patients with 2+ fractures had significantly higher incidence of DPN (but not other complications) even with adjustment for glycemic control and disease duration. IV	Leanza et al. [85]
25	M/F	50–75	294	T2D	Neuropathy	UKST, ankle reflex, pin-prick, VPT, temperature, PROs, NCV	Fracture	Y	After adjustment for age, sex, disease duration, BMI, DPN had strongest association with fracture. III-3	Kim et al. [86]
26	M/F	16–70	498,617	T1D/T2D	Neuropathy	ICD codes	Fracture	N	After adjustments, no increased risk of fracture in T1D or T2D (separate) due to DPN. III-3	Vestergaard et al. [87]
27	M/F	64 ± 13	105	T1D/T2D	Neuropathy	Monofilament	Healing	Y	DPN was associated with higher rate of healing complications. III-3	Wukich et al. [88]
28	M/F	37–80	322	T1D/T2D	Neuropathy	ICD codes	Healing	Y	After adjustments, peripheral neuropathy had the strongest association with bone healing complications. III-2	Shibuya et al. [89]

*M/F* male/female, *VPT* vibration perception threshold, *QST* quantitative sensory testing (temperature, vibration, pressure), *NCV* nerve conduction velocity, *HRV* heart rate variability (deep breathing and/or lying-to-standing), *MNSI* Michigan Neuropathy Screening Instrument, *UKST* United Kingdom Screening Test, *EMG* electromyography, *PROs* patient-reported outcomes (symptoms), *ICD* International Classification of Diseases, *DEXA* dual-energy X-ray absorptiometry, *DPA* dual-proton absorptiometry, *QUS* quantitative ultrasound, *pQCT* peripheral quantitative computed tomography, *HR-pQCT* high-resolution peripheral computed tomography, *NR* not reported.

^a^National Health and Medical Research Council Evidence Hierarchy according to Aetiology research questions: IV, cross-sectional study; III-3, case-control study; III-2, retrospective cohort study; III-1, all or none; II, prospective cohort study; I, systematic review of level II studies

Within the clinical literature, the presence of neuropathy, retinopathy, and nephropathy are often considered together as a proxy for MVD in diabetes [4, 7, 18, 20••, 71]. Though this oversimplifies the pathogenesis of these disorders, the MVD model provides an excellent starting point to understand the relationship between diabetic complications and skeletal disease. To date, 15 clinical studies implicate a role for neuropathy alone, or neuropathy within the context of MVD, in higher fracture risk or reduced bone quality in T1D and T2D [4, 7, 20••, 66–71, 80, 81, 83••, 84–86] (Table 1). The most common explanation for the impact of MVD on fracture risk is loss of proprioception (neuropathy) and vision (retinopathy), increasing the likelihood of falls [90]. In addition, reduced physical ability and functional limitations develop alongside motor neuropathy and may contribute to reduced bone quality and increased fall risk in T1D and T2D [24•, 91, 92]. However, while a correlation with increased fall frequency has been confirmed [90, 93, 94], this view of diabetic complications, particularly DPN, likely underestimates the scope of its effects on bone health. DPN has been associated with reduced BMD or higher fracture risk in patients with T1D in seven separate clinical studies [66–70, 84, 85]. Isolated cohorts of T2D are much less frequent [18, 20••, 74, 80, 83••, 86] (Table 1), but one retrospective cohort of 2,798,309 older male veterans found that as much as 21% of the increase in fracture risk in T2D was explained by neuropathy, making it the highest contributing factor of all complications examined [83••]. In total, ten clinical studies have found strong associations between DPN, increased fracture risk, and/or reduced BMD [66–70, 81, 83••, 84–86]. Yet, an almost equal number find evidence to the contrary [72–79, 82, 87]. In some cases, multivariate models correcting for glycemic control and disease duration—factors often used to explain associations between neuropathy and fracture risk—reduce the impact of DPN below significance [76, 87]. Hence, it remains unresolved whether neuropathy simply co-evolves with diabetic bone disease due to similar pathological mechanisms (i.e., both serve as markers for disease duration or glycemic control), or if a causal relationship exists.

In addition to BMD and fracture, other measures of bone quality (e.g., microarchitecture, trabecular bone score) may relate to neuropathy in both T1D and T2D [28]. Two comprehensive studies by Shanbhogue et al evaluated the impact of MVD on bone density and microarchitecture using HR-pQCT in patients with T1D [7] and T2D [20••]. These studies are notable since they include comparisons between diabetic and non-diabetic MVD− patients and to non-diabetic MVD+ controls. Relative to age-matched, non-diabetic patients with MVD, T1D patients with MVD have significantly lower trabecular and cortical volumetric BMD, reduced cortical thickness, and increased periosteal circumference; when compared to diabetic patients without MVD, T1D is associated with additional decreases in trabecular volumetric BMD and thickness in the presence of microvascular complications [7]. T2D patients with MVD have more subtle changes in microarchitecture including trends toward increased cortical porosity, decreased cortical thickness, decreased cortical volumetric BMD, and, conversely to patients with T1D, increased trabecular bone mass [20••]. These changes are not present in the diabetic MVD− groups when compared to age-matched, non-diabetic MVD− controls. These studies suggest that adult patients with longstanding T1D (4 to 54 years) or T2D (10 to 17 years) have relatively preserved bone microarchitecture and, in the case of T1D, normal predicted bone strength in the absence of MVD [7, 18, 20••]. The underlying mechanisms associating MVD complications and diabetic bone disease remain unknown and controversial, particularly in regard to neuropathy. Current research is focused on defining whether these associations have positive predictive value for fracture, or beyond this, a direct impact on impaired bone health.

Bone healing represents another approach to characterize the effect of DPN on skeletal health. In one retrospective study, bone healing complications after foot or ankle surgery were observed with higher prevalence in diabetic patients with neuropathy (half of patients); in fact, DPN was considered the most important factor in determining bone healing complications such as nonunion, delayed union, or malunion [89]. A similar investigation observed that 76% of diabetic patients with DPN sustained post-operative healing complications after surgical treatment for ankle fracture [88]. However, neither study differentiated between T1D and T2D and the method of neuropathy diagnosis was limited to sensory neuropathy or unclearly stated.

Indeed, a key limitation within the current body of literature is a lack of consistency and comprehensiveness of neuropathy assessments and diagnoses. Comprehensive neuropathy assessments necessitate consideration of sensory, autonomic, and motor fibers. Some assessments, including nerve conduction velocity (NCV), monofilament tests, reflex tests, and vibration perception tests (VPT), are better suited for detecting large nerve fiber dysfunction (sensory and motor); others, including thermal tests, pinprick tests, and autonomic evaluations, are more indicative of small fiber involvement (autonomic and sensory) [48]. Assessments vary widely, especially in cross-sectional studies; only three have clearly included separate assessments for large and small fiber neuropathy [66, 82, 86]. While sensory symptoms are the most common presentation of DPN, it is often concomitant with autonomic and motor neuropathy, both of which are relevant to bone. Only two clinical studies have attempted to isolate the impact of autonomic neuropathy on bone metabolism in T1D. The first concluded that no relationship exists; however, the study was limited by sample size and diagnostic tools, and there is growing evidence that autonomic neuropathy reduces BMD in T1D, at least in the trabecular compartment [70, 75]. In addition, diabetic motor neuropathy may contribute to muscular atrophy in T1D [50], reducing muscle-mediated bone loading and myokine secretion that may be important to bone metabolism [95].

## Parsing the Potential Trifold Contributions of DPN to Diabetic Bone Disease

An association between decreased bone health and neuropathy, independent of other MVD complications, is emerging. As eluded to in the introduction, relationships between nerve and bone health are generally segmented into three major categories: neurotraumatic, neurotrophic, and neurovascular (reviewed in [1]). The potential implications for each of these in the setting of diabetic bone disease will be discussed below.

1. Neurotraumatic contributions: impact of altered loading and microtrauma on bone

When it was first introduced by Volkman and Virchow to explain the pathogenesis of Charcot neuroarthropathy, the German neurotraumatic theory attributed neuropathic bone and joint degeneration to abnormal loading and unrecognized trauma or microfracture due to loss of proprioception and general sensation [96]. Changes in walking gait due to sensory and motor neuropathy and skeletal muscle atrophy have been noted in clinical studies of diabetic patients even without neuroarthropathy [97–99]. Resulting gait changes have the potential to subject bone to abnormal stresses, perhaps affecting bone quality even at mechanoresponsive sites far away from the joint [100]. Correlations between gait-related changes in skeletal loading, DPN, and focal changes in bone microarchitecture or mineral density have not yet been established, but represent an interesting area of future investigation.

2. Neurotrophic contributions: cellular and molecular mechanisms linking neuropathy and bone in diabetes

The bone and bone marrow are innervated by small-diameter sensory and autonomic nerves, and a growing body of literature suggests that neurotrophic regulation of bone metabolism may play a role in skeletal homeostasis [1]. Bone maintenance, or remodeling, is carried out by basic multicellular units of osteoblasts and osteoclasts. Osteoclasts initially excavate a resorption space which is then filled with new bone by osteoblasts, creating osteons within the cortex and hemi-osteons on the endocortical surface or within the cancellous bone compartment [100, 101]. Dysregulation of this process can result in decreased cortical and trabecular bone, or increased cortical pore number and size, as has been observed in patients with T1D and T2D, respectively. Within the cortical bone, blood vessels and their associated nerves are recruited and extend through the newly formed osteon at a rate comparable to that achieved by the osteoclast cutting cone [101]. There is also evidence that nerve profiles are generally increased near active remodeling surfaces in human bone, particularly in the cortex [102••]. The proximity of nerve fibers to bone cells suggests the possibility of direct regulatory actions on osteoblasts, osteoclasts, and osteocytes.

In a meta-analysis of clinical studies, circulating osteocalcin was the only biomarker that was consistently reduced in both T1D and T2D regardless of sex or age, reflecting common suppression of osteoblast recruitment and/or function across patient groups [103]. Regulation of osteoclasts may also occur, particularly in post-menopausal individuals; however, osteoclast biomarkers are highly heterogeneous between studies and patients [103]. In the presence of diabetic neuropathy, changes in local or systemic neurotransmitters due to altered production, impaired axonal transport, and/or loss of free nerve endings may contribute to these processes. For example, serum levels of calcitonin gene-related peptide (CGRP) and substance P, two sensory neuropeptides with osteoanabolic potential, were found to be reduced by ~ 50% in adults with diabetes [104]. In addition, plasma catecholamine concentrations have been shown to be elevated in untreated diabetics [105] or reduced in patients with diabetic adrenergic neuropathy [106], suggesting potential for adrenergic neurotransmitter-mediated contributions to bone metabolism at different stages of disease.

It is also worth noting that osteocytes and bone marrow adipocytes represent alternative mediators of the coupling between nerve and bone in diabetes. Little is known about neurotrophic regulation of osteocytes; however, load-induced bone formation, commonly thought to be a key function of osteocytes, is hindered in animal models of diabetes [107–109], and a potential role of nerves in mediating load-induced bone formation is under investigation [110–112]. Bone marrow adipocytes are also increased in a subset of patients with diabetes (reviewed in [113]). Three-dimensional electron microscopy imaging has revealed a close association of bone marrow adipocytes with sinusoidal vessels and sympathetic nerves, in close proximity to osteoblasts in mice, suggesting the potential for shared nerve-adipocyte-osteoblast regulatory pathways [114•].

3. Neurovascular contributions: changes in blood flow governing diabetic bone metabolism

Within human bone, ~ 95% of nerves are associated with blood vessels [102••] and vasculogenesis is a key component of both bone modeling and remodeling [101]. Sympathetic adrenergic nerves mediate local vasoconstriction. By contrast, sensory neuropeptides CGRP and substance P are highly potent vasodilators [115]. CGRP and substance P can also promote angiogenesis [116, 117] and augment vascular permeability [118]. Thus, dysregulation of vascular perfusion of bone due to autonomic and sensory dysfunction in diabetic neuropathy represents another avenue through which DPN may contribute to diabetic bone disease. This is commonly referred to as the neurovascular theory. In addition, osteoclast and osteoblast progenitors are often derived from the circulation and the perivascular niche, respectively. Thus, diabetes-associated vascular disruptions may reduce access to these crucial progenitor depots.

Clinical studies which monitor peripheral limb blood flow via laser Doppler or scintigraphy have been performed in patients with diabetes; however, they generally have small sample sizes, contradictory results, and focus exclusively on Charcot neuroarthropathy. Three-phase bone scintigraphy of T2D patients with and without neuropathy was thought to reveal increased blood flow to bone even before the development of disease [119]. This could indicate a loss of sympathetic tone in the peripheral vasculature supplying the limb; however, a later study was unable to detect changes in venoarteriolar sympathetic axon reflex in association with neuroarthropathy [120], and another suggested that sympathetic neuropathy may actually protect against the development of neuroarthropathy [121]. Consensus has yet to be reached on a consistent relationship between vascular function and the development of diabetic bone disease. The challenge may lie in the changing nature of this relationship as the disease progresses and as additional complications arise. While sympathetic neuropathy in the peripheral vasculature may lead to dilation of blood vessels, vascular calcification and microangiopathy may also reduce effective sympathetic control of vascular tone. Longitudinal investigations and identification of proper assays are needed to evaluate this relationship both in neuroarthropathy and diabetic bone disease.

## Prospective Areas of Research and Future Directions

Progressive characterization of the interplay between DPN and bone disease could play a pivotal role in advancing the field of neuroskeletal biology. Moving forward, clinical investigations will benefit from enhancing current study designs and expanding areas of research to promote more comprehensive and consistent neuropathy assessments, for example, inclusion of autonomic and motor modules in addition to sensory assays for both large and small fibers. These enhancements would not only improve neuropathy detection but may also provide adequate homogeneity for future meta-analysis. To date, the Michigan Neuropathy Screening Instrument (MNSI) has been one of the most widely applied chairside instruments for standardized clinical evaluation of diabetic peripheral sensory neuropathy [122]. Advanced techniques including electrophysiology and vascular studies are also possible but require expertise and equipment that is not widely available. To overcome this, an expansion of relatively non-invasive neuropathy diagnostic tools with supplemental or improved detection capabilities could reinvigorate clinical studies. For example, sudomotor tests (SudoScan) and corneal confocal microscopy are novel non-invasive detectors for peripheral autonomic and small fiber neuropathy, respectively [123, 124]. Most critically, prospective cohort studies in T1D and T2D with careful longitudinal assessments of both DPN and bone disease, starting close to onset, will be needed to establish the temporal relationship between these associated complications.

Paradigms for parsing the trifold contributions of DPN are largely undeveloped and represent a novel research frontier. Cross-sectional kinematic studies with bone assessments in diabetic patients with and without DPN may be an appropriate starting point for neurotraumatic contributions. Histological investigations of human diabetic bone may reveal the presence or absence of local neurotrophic relationships between skeletal nerves and bone cells in DPN. Ultimately though, developing this framework will require innovative collaborations between neurobiologists, bone biologists, and engineers to identify novel methods to specifically assess neurovascular function in bone and local cellular interaction between nerves and bone cells.

## Therapeutic Considerations for DPN and Diabetic Bone Disease

**Fig. 1:**
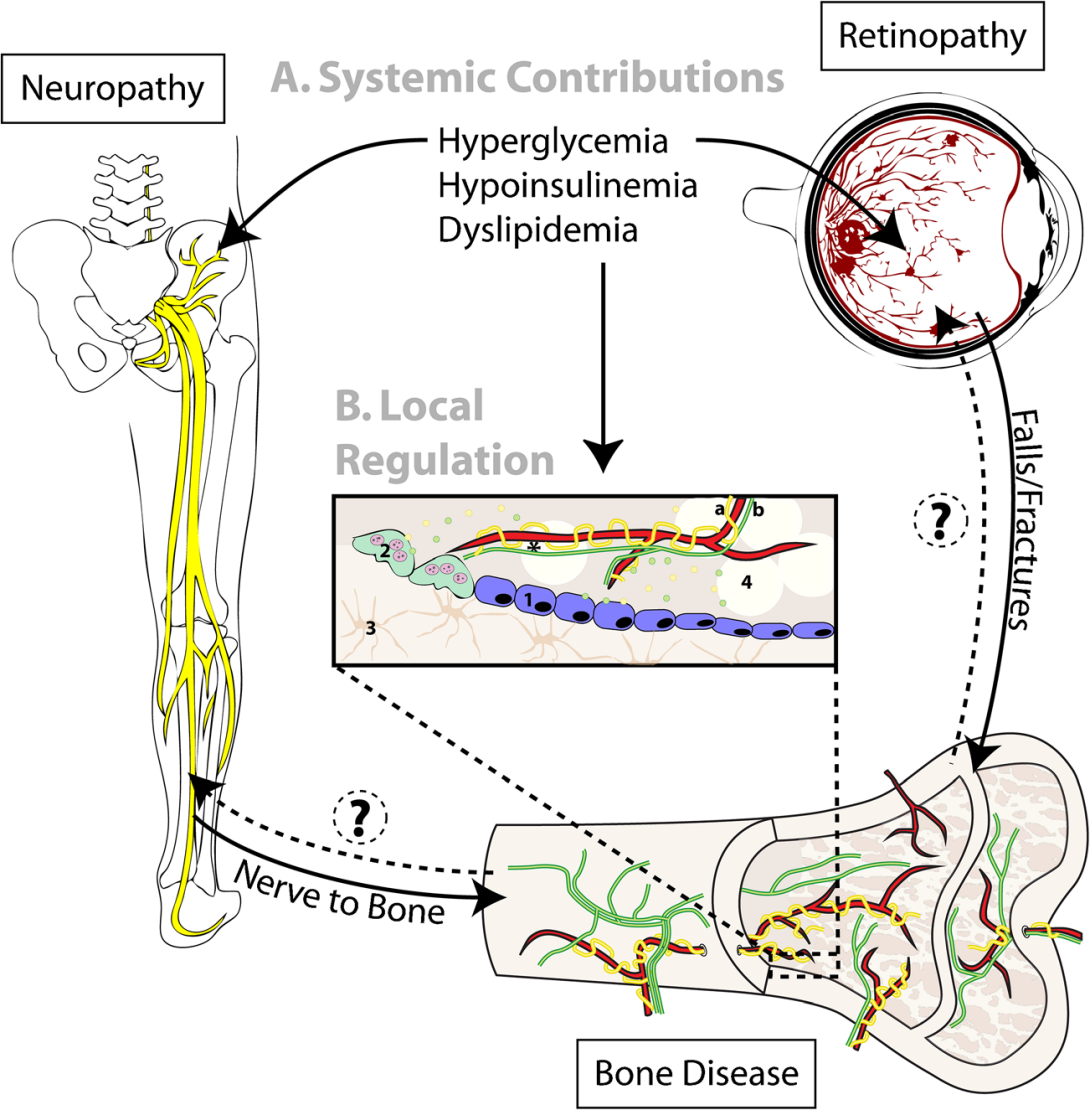
Systemic and local relationships between diabetic neuropathy and bone health. **a** T1D and T2D result in hyperglycemia, hypoinsulinemia (T1D and some T2D), and dyslipidemia that impact multiple peripheral organ systems. These changes, among others, are part of a complex multifactorial set of systemic mediators that promote development of diabetic complications including peripheral neuropathy, retinopathy, and bone disease. Complications such as retinopathy can indirectly influence bone outcomes by increasing the risk of falls and fractures. Similarly, development of neuropathy can cause muscle weakness, altered gait, and impaired skeletal loading in addition to increasing risk of falls and fracture. **b** Beyond this, the skeleton is locally innervated by both (a, yellow) sympathetic adrenergic and (b, green) sensory peptidergic nerves. Secreted neurotransmitters have the capacity to act on surrounding cells including (1) osteoblasts, (2) osteoclasts, (3) osteocytes, and (4) bone marrow adipocytes. In addition, these neurotransmitters play a key role in regulating local vascular tone and permeability (*). Altogether, this provides many potential avenues for local regulation of bone metabolism and quality, which may be altered in the presence of neuropathy. (?) Emerging evidence also suggests that neural regulation of the bone marrow may stimulate release of circulating progenitors which promote tissue repair at distant sites. Thus, when present within bone, diabetic peripheral neuropathy may impair progenitor release, promoting further deterioration of both nerves (neuropathy) and vessels (retinopathy). Moving forward, both clinical and basic research is needed to establish which of these relationships are necessary and/or sufficient for destabilization of bone in patients with diabetes. This will promote optimization of therapeutics and interventions, promoting skeletal health across the lifespan

DPN has great potential to influence bone metabolism and fracture risk though a diverse array of systemic and local mechanisms (Fig. 1). Conversely, there is emerging evidence in rodents and humans that neural regulation of the skeleton may be critical for maintenance of peripheral tissues in diabetes [125, 126]. Specifically, damage to nerves within the bone marrow may restrict autonomic-mediated release of circulating progenitor cells [125, 126], potentially contributing to further progression of diabetic complications including retinopathy and neuropathy [126, 127]. This vicious cycle of disease provides an important context in which to consider interventions for patients with diabetes. It suggests that common strategies and therapeutics which simultaneously maintain or re-establish both nerve and bone health, and their relationship with one another, are needed and may subsequently have a positive impact on progression of other diabetic complications such as retinopathy. This, in turn, may limit falls and help to further decrease fracture. This integrated approach has great potential to reduce overall fracture risk and promote health span of aging diabetic individuals. These considerations may be especially important in adolescent-onset diabetes with longer disease duration. As much as 7% of adolescents with T1D and 22% of adolescents with T2D already have symptoms of neuropathy [43••]. Thus, early intervention may be critical to delay the onset of both nerve damage and skeletal disease in diabetes.

## Conclusions

Peripheral neuropathy can have devastating consequences on quality and quantity of life. Due to its prevalence in both diabetic and non-diabetic populations, the relationships between nerves and their end-target organs merit comprehensive investigation. At this time, a compelling association is emerging between diabetic neuropathy and bone disease, but evidence is still forthcoming regarding the direct effects of diabetic neuropathy on bone or the utility of DPN as a predictor of skeletal fragility and fracture. To the authors’ knowledge, no prospective cohorts with follow-up bone and nerve assessments exist within the current body of literature. In addition, variance in bone and nerve assessments and inclusion of mixed diabetic (T1D and T2D) cohorts may contribute to study heterogeneity (Table 1). While most associations between neuropathy and bone disease have been reduced to increased fall frequency, a trifold model of the nerve-bone axis may serve as a guide to study neuropathic contributions to skeletal fragility in diabetes. In addition to greater methodological cohesion, novel experimental approaches are needed to systematically interrogate this multifaceted relationship and to clarify the role of peripheral neuropathy as a component of skeletal disease in diabetes.
